# Laryngeal Masks in Neonatal Resuscitation—A Narrative Review of Updates 2022

**DOI:** 10.3390/children9050733

**Published:** 2022-05-17

**Authors:** Srinivasan Mani, Joaquim M. B. Pinheiro, Munmun Rawat

**Affiliations:** 1 Pediatrics, University of Toledo, Toledo, OH 43606, USA; 2 Pediatrics, Albany Medical Center, Albany, NY 12208, USA; pinheij@amc.edu; 3 Pediatrics, University at Buffalo, Buffalo, NY 14260, USA; munmunra@buffalo.edu

**Keywords:** laryngeal masks, infant, neonate, resuscitation

## Abstract

Positive pressure ventilation (PPV) is crucial to neonatal cardiopulmonary resuscitation because respiratory failure precedes cardiac failure in newborns affected by perinatal asphyxia. Prolonged ineffective PPV could lead to a need for advanced resuscitation such as intubation, chest compression, and epinephrine. Every 30 s delay in initiation of PPV increased the risk of death or morbidity by 16%. The most effective interface for providing PPV in the early phases of resuscitation is still unclear. Laryngeal masks (LMs) are supraglottic airway devices that provide less invasive and relatively stable airway access without the need for laryngoscopy which have been studied as an alternative to face masks and endotracheal tubes in the initial stages of neonatal resuscitation. A meta-analysis found that LM is a safe and more effective alternative to face mask ventilation in neonatal resuscitation. LM is recommended as an alternative secondary airway device for the resuscitation of infants > 34 weeks by the International Liaison Committee on Resuscitation. It is adopted by various national neonatal resuscitation guidelines across the globe. Recent good-quality randomized trials have enhanced our understanding of the utility of laryngeal masks in low-resource settings. Nevertheless, LM is underutilized due to its variable availability in delivery rooms, providers’ limited experience, insufficient training, preference for endotracheal tube, and lack of awareness.

## 1. Introduction

Perinatal asphyxia in newborns results in the development of respiratory failure before the onset of cardiac failure. The typical sequence of respiratory failure preceding cardiac failure in asphyxiated newborns differs from that of adults, where circulatory and respiratory failure co-occur [1]. This underlies the critical difference between the resuscitative efforts of newborns compared to older children and adults. Positive pressure ventilation (PPV) is more crucial to newborn resuscitation than in adult cardiopulmonary resuscitation (CPR), where chest compression plays a critical role. The inability to provide effective PPV promptly to newborn infants affected by asphyxia has been shown to result in death and disability. Every 30 s delay in initiation of PPV increased the risk of death or morbidity by 16% in an observational study conducted in a rural hospital in Tanzania [2].

The most effective interface for providing PPV in the early phases of resuscitation is still unclear [3]. Face mask (FM) is the most commonly used interface for delivering PPV in the delivery room. The problems associated with FM PPV are mask leak, airway obstruction related to variable operator skills, and trigeminocardiac reflex (TCR). All the listed problems are more pronounced in preterm than term infants [4,5]. TCR, more specifically the peripheral TCR, can occur due to the stimulation of maxillary (V2) and mandibular (V3) divisions of the trigeminal nerve activated by the application of an FM, which leads to stimulation of brainstem nuclei which in turn can cause apnea, bradycardia, and hypotension through reflex vagal action [6]. Subgroup analysis of a randomized trial comparing FMs in infants > 34 weeks found that the application of FM for newborn stabilization resulted in apnea in 11% of the infants. This physiological response, considered to be mediated by peripheral TCR, was more pronounced with the first application of the FM [7]. In the absence of a respiratory function monitor during resuscitation, airway obstruction and mask leak are often unrecognized. Delay in corrective measures could lead to a delay in the effective delivery of PPV [8]. Prolonged ineffective PPV results in a need for advanced resuscitation such as intubation, chest compression, and epinephrine [9].

The interfaces studied as alternatives to FM for delivering PPV to depressed newborns in the initial stages of resuscitation are the laryngeal mask (LM), nasal prong, and nasopharyngeal tube. Although the nasal or nasopharyngeal interface showed promise with a theoretical advantage of reducing the effect of peripheral TCR, recent studies did not find convincing evidence. A recent retrospective matched-pairs study that compared the use of binasal prongs and FM for resuscitation in preterm infants < 32 weeks found no difference in the rate of occurrence of apnea and bradycardia. The authors of this study hypothesize that binasal prongs could still trigger the TCR by stimulating the trigeminal receptors inside the nose or over the maxillary region (V2) [10]. A meta-analysis including five RCTs conducted over a 12-year period compared the nasal prongs or nasopharyngeal tube with FM for delivery room resuscitation in infants born < 37 weeks. Four out of five RCTs included very preterm infants. The meta-analysis found no difference in the in-hospital mortality between nasal interface and FM. Although there was a reduction in the rates of delivery room intubation and chest compressions with the nasal interface, this advantage was not sustained at 72 h. Additionally, there was an increased risk of severe grade IVH with the nasal interface than FM [11].

LM as an interface to deliver PPV for initial stabilization of the newborn has been studied for the last three decades. A recent systematic critical appraisal of the literature found that LM is a safe and more effective alternative to FM ventilation in neonatal resuscitation [12,13].

This article aims to provide a narrative review of the literature regarding the use of LM in neonatal resuscitation. We will highlight the evolution of the LM devices and the adoption of their use in neonates over the years. We will synthesize the current evidence regarding the use of LM in various phases of neonatal resuscitation, emphasizing the recent updates. We will discuss the existing knowledge gaps and suggest future directions on this topic.

## 2. Methods

We have summarized the research findings from an extensive literature review utilizing key terms in multiple databases, including MEDLINE via PubMed, EMBASE, Web of Science, and the Cochrane library. We used the MeSH terms “Laryngeal Masks” AND “Infant OR Newborn” AND “Resuscitation” for our PubMed search. A similar search strategy was adopted to suit EMBASE, Web of Science, and the Cochrane library. We searched the database up until December 2021.

## 3. Discussion

### 3.1. Historical Perspectives

LM was introduced as a new airway device, with intermediate usability between FM and endotracheal tube (ETT). A pilot study of the successful use of this device in 23 patients was reported in 1983 by A.I.J Brain [14]. An observational study of LM in infants during elective minor surgery for anesthesia established the feasibility and safety of its use in 1992 [15]. The use of LM as an alternative to bag-mask ventilation in neonatal resuscitation was first studied in 1994 [16]. The first clinical trial comparing face mask use vs. LM in neonatal resuscitation was published in 2005 and showed LM to be an easy, safe, and effective alternative to FM [17]. Subsequently, the first clinical trial reported in 2008 compared the FM, LM, and ETT, showing that the LM was effective and the fastest method in achieving successful neonatal resuscitation [18]. A randomized clinical trial comparing a second-generation LM having a gastric drain tube with an FM showed that LM is more efficacious than FM ventilation in preventing endotracheal intubation during neonatal resuscitation at birth [19]. In 2015, the Neonatal Resuscitation Program (NRP) recommended LM as an alternative to ETT in situations where resuscitators “cannot intubate and cannot ventilate” [20]. The updated systematic review of the Cochrane database in 2018 concluded that LM is more efficacious than BVM and comparable to ETT as an airway device during delivery room resuscitation of term and late preterm newborns [13].

### 3.2. Classification of Supraglottic Airway Devices

**Figure 1:**
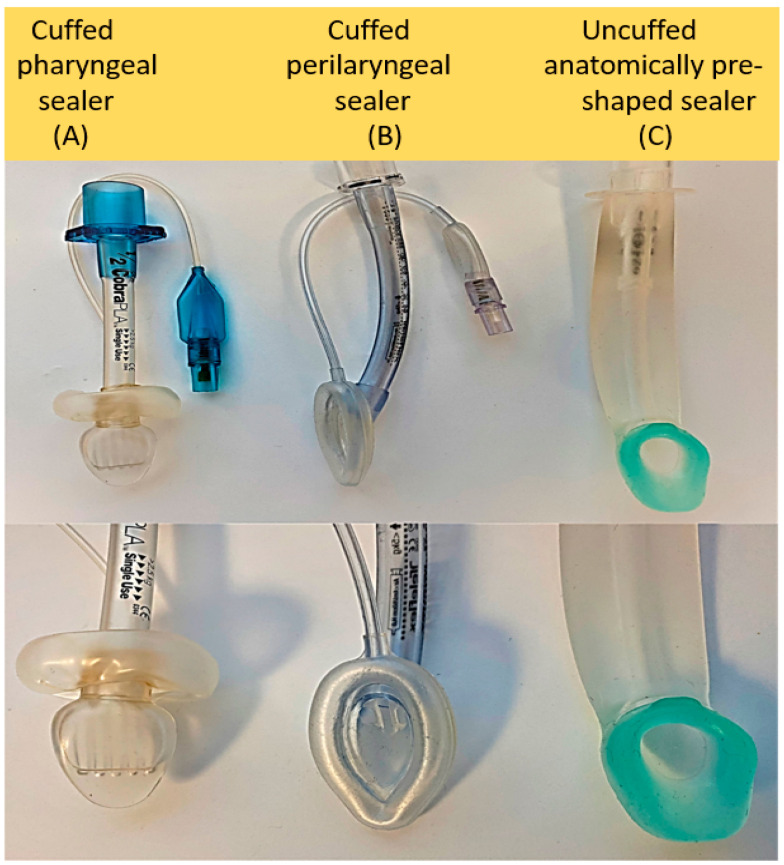
Neonatal-sized samples of the three archetypal devices, namely (**A**) Cobra Perilaryngeal Airway, (**B**) Laryngeal Mask Airway Unique, and (**C**) i-gel.

Supraglottic airway or extraglottic airway devices (SGADs) are a class of airway devices placed outside the glottis to provide airway access for positive pressure ventilation as an alternative to the face mask and endotracheal tube. Approximately 25 different SGADs are available in current clinical practice [21]. They are classified into three major categories based on their sealing mechanism, including (1) cuffed pharyngeal sealers, (2) cuffed perilaryngeal sealers, and (3) cuffless anatomically preshaped sealers [22] (see Figure 1). These three classes are divided into single-use and reusable devices. Different types of LM come under the cuffed perilaryngeal sealing device, and i-gel, a device more recently studied in neonatal resuscitation, is classified under the cuffless preshaped sealing device. LM has also been classified into first-generation and second-generation LM with gastric access, intubating LM, and upper gastrointestinal endoscopic LM. As specialized features have been added to existing designs, simple classification schemes are less useful in practice; instead, selection of models should be based on characteristics which maximize clinical efficacy in the target population and usability by providers.

### 3.3. LM and Its Effect on Respiratory Mechanics

Ventilatory compromise can be caused by gastric insufflation due to LM use. Studies using LM in anesthetized pediatric patients beyond infancy have shown that gastric insufflation with an adequately positioned LM is less than that of an FM and comparable to an uncuffed ETT [23,24]. The reduced dead space of LM compared to the FM is advantageous. However, a recent sub-study of the NEOSUPRA trial found that the mask leak was similar between i-gel (cuffless SGAD) and FM. The expired tidal volume, peak inspiratory pressure, and inspiratory tidal volume were similar between the FM and i-gel [25]. Airway resistance is inversely related to the fourth power of the airway radius. The resistive load offered by the LM is less than that of the ETT because the internal diameter of the ETT in the commonly used neonatal sizes is narrower than the LM. LM allowed the use of lower PIP than ETT in anesthetized pediatric patients in the 1–7 year age group by reducing airway resistance and increasing dynamic lung compliance [26]. However, a study conducted in adults undergoing anesthesia found that if the larynx is included in calculating airway resistance, the LM’s resistance was equivalent to ETT [27]. A comparison of resistive load among the different generations of LM in anesthetized adults showed that the proper positioning of the device with a good seal is a determining factor [28]. The studies looking into the respiratory mechanics with LM were carried out predominantly in the pediatric population under anesthesia with age > 1 year. The effect of LM on respiratory mechanics in a partially fluid-filled lung at the time of birth has not been systematically studied so far.

### 3.4. LM—A Peri-Laryngeal Sealing Device

**Figure 2:**
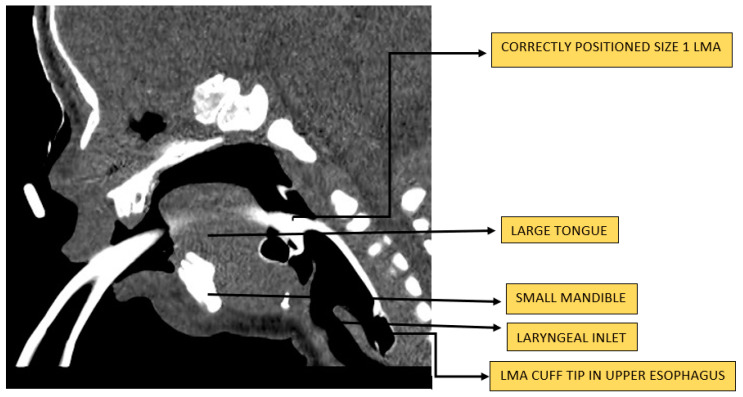
CT image of a size 1 LMA^®^ in situ in a neonate with severe micrognathia and congenital upper airway obstruction.

LMA Classic^TM^ size 1 is the LM most studied in neonates. It can be used in infants up to a weight of 5 kg. Several manufacturers make a variety of size 1 LMs, but there is no manufacturing standard for size designations, and both mask and ventilatory pathway dimensions vary significantly by model within the same nominal size. Devices similar to the LMA Classic^TM^ are made of medical-grade polyvinyl chloride (PVC) or silicone. Silicone-based LMs provide more elasticity to conform to the anatomy and provide higher oropharyngeal seal pressure without the risk of phthalate exposure from PVC. Some of the silicone-based LMs are reusable up to 60 times. Oropharyngeal leak pressure (OLP), also referred to as airway sealing pressure or airway leak pressure, is an important parameter that reflects the device’s correct placement and adequate airway seal [29]. In the US, the single-use disposable LM made of medical-grade PVC is the most commonly available type. It consists of three main parts: a ventilatory pathway with an internal diameter of 5.3 mm, an elliptical mask-shaped inflatable cuff on one end, and a 15 mm male connector on the other end [30]. It contains an inflation line with a one-way pilot valve to inflate the cuff. The manufacturer recommends inflation of up to 4 mL of air. The fully inflated cuff of a correctly placed LM occupies the hypopharynx creating an airtight perilaryngeal seal with its lumen facing the laryngeal inlet (see Figure 2).

### 3.5. A Second-Generation LM

Some second-generation LMs have a drain tube that sits over the esophagus, enabling users to vent the stomach. An orogastric tube can be inserted through the esophageal drain tube to overcome gastric insufflation from air leaks during PPV with LM. The newer LM devices allow the use of PIP higher than 20 cm H_2_O because of their higher OLP [31]. LM supreme and the i-gel are the newer LM types well studied in the neonatal population, specifically in delivery room resuscitation [19,32]. LMA Proseal ^TM^ is another second-generation LM studied as a conduit for endotracheal intubation in manikin-based simulation experiments [33].

### 3.6. LM Use in Current Neonatal Practice and Perceptions

LM is recommended as an alternative airway device for the resuscitation of infants ≥34 weeks by the International Liaison Committee on Resuscitation (ILCOR), and it has been adopted by various national neonatal resuscitation guidelines, including the United States, Europe, and Australia. Nevertheless, there is a great degree of variability in the practical adoption of these recommendations. A survey using an online questionnaire including 29 tertiary care NICUs within the Australia New Zealand Neonatal Network showed that LM was unavailable in 33% of the centers. In centers where the LM was available, 60% of the staff lacked adequate skills to use them on infants [34]. A single-center simulation study performed at a level IV regional perinatal center in the US compared the proficiency of LM with ETT insertion among NRP providers of various experience levels. The study found that the NRP providers had a higher failure rate with LM and were not confident enough to use the device during resuscitation [35]. A recent online questionnaire-based observational study carried out in another US tertiary care NICU found that LM is underutilized due to providers’ limited experience, insufficient training, preference for endotracheal tubes, and lack of awareness [36]. Conversely, at some tertiary care NICUs across the US, most neonatologists, mid-level providers, respiratory therapists, fellows, and even pediatric trainees have had experience in neonatal LM use, largely stemming from involvement in surfactant administration studies [37].

### 3.7. Insertion Technique and Training

AHA NRP recommends the standard insertion technique for LM use in neonates [38]. The resuscitator stands at the head end with the infant in a sniffing position in the standard technique. In the case of a cuffed LM, LM is held by the airway tube with the closed bottom of the mask facing the infant’s palate with a fully deflated cuff. The device is glided over the tongue downward and backward, following the palate’s contour with a gentle push. In spontaneously breathing patients, connecting a CO_2_ detector to the LM before insertion facilitates monitoring for airway obstruction during the insertion process. When resistance is reached, the airway tube with a CO_2_ detector is held in place—otherwise, unanesthetized neonates can expel the device. Cuffed LMs for neonates (size 1) are inflated with 3–5 mL of air based on the manufacturer’s recommendation while observing the CO_2_ signal for possible development of airway obstruction, which might necessitate slight repositioning of the LM usually by withdrawing a few millimeters; then, PPV is initiated. In neonates, particularly in the delivery room, existing upper airway moisture renders the application of water-based lubricant to the LM cuff unnecessary.

There are several alternative techniques of LM insertion mainly studied in adult patients. Variations of the standard technique include inserting the LM with the cuff partially or fully inflated as against deflated cuff in the standard technique [39]. There are two rotational techniques, namely 180° and 90° techniques [40]. In the 180° technique, an LM with a fully deflated cuff is inserted back-to-front like a Guedel airway and then rotated counterclockwise through 180° as it is pushed into the hypopharynx. The 90° rotational technique is similar to the 180° technique except that after the LM device with a fully deflated cuff is inserted laterally into the mouth, it is rotated counterclockwise through 90° and advanced and straightened out in the hypopharynx. These rotational techniques have been shown to be better than the standard technique in randomized trials in children and adults during anesthesia [41,42,43]. Nevertheless, apart from the standard technique, none of these techniques have been studied in newborn infants during resuscitation.

Healthcare workers can be easily trained in LM insertion with a brief ≤15 min manikin-only training [44]. This is in contrast to the long duration and repetitive training required to master the skills of endotracheal intubation and FM ventilation. In a web-based national survey conducted in the UK to assess the experience and training in endotracheal intubation among pediatric trainees, neonatal trainees, and neonatal nurse practitioners (ANNPs), only 18% of the 646 respondents felt completely confident at intubation [45]. At least 40 intubations are required to achieve proficiency [46]. However, this number is challenging to achieve during pediatric training, even though endotracheal intubation is a mandatory skill. Less than 50% of general pediatric trainees and ANNPs reported performing >20 neonatal intubations. FM ventilation is a much simpler skill, but avoiding mask leaks and airway obstruction is difficult without regular practice, especially without a respiratory function monitor [47,48,49]. Inter-individual variability in skill retention is much lower with LM. Brief training of study participants aimed at comparing ease of insertion and efficacy of ventilation of various second-generation LM has shown high rates of success in the first attempt ranging from 87.5% to 97.5% [50,51].

### 3.8. LM during Initial Stabilization

The use of LM for positive pressure ventilation in the initial stabilization of an asphyxiated newborn has been studied both as a first-line alternative to FM and a rescue interface with ETT as a comparator.

#### 3.8.1. LM as Primary PPV Interface

In the most recent Cochrane database meta-analysis, seven RCTs were included for quantitative synthesis of evidence, of which four studies compared LM with FM [17,19,52,53], and one study had a three-arm comparison between LM and FM and ETT [18] (see Table 1). The primary outcome studied in most of these studies was successful resuscitation without the need for intubation. The review concluded that very low to moderate quality evidence suggests that LM can achieve adequate ventilation in neonates and is more effective than FM in resuscitation settings. Seven outcomes were analyzed systematically. They were: (1) failure of the primary modality of resuscitation, (2) need for intubation, (3) time to spontaneous breathing, (4) ventilation time, (5) Apgar score ≤ 7 at 5 min, (6) admission to NICU, (7) death or hypoxic-ischemic encephalopathy (HIE). The analysis favored the use of LM in all but one outcome. Death or HIE was not different between the two ventilation interfaces. However, only two studies in the analysis reported this outcome with a 95 (LM) vs. 96 (FM) sample size.

Two RCTs that compared LM with FM were not included in the Cochrane review [32,54]. The trial by Mathai et al. was carried out with a sample size of 67 infants and evaluated the duration of PPV until spontaneous breathing as the primary outcome. They found that the infants in the LM group required a significantly shorter duration of PPV before spontaneous breathing was established (95.31 s (23.22 s) vs. 180.86 s (37.83 s) (*p* = 0.024)) and a reduced need for endotracheal intubation (15.6% vs. 34.3%).

The study by Pejovic et al. in 2020 was a large superiority trial of LM versus FM with a sample size of 1154 infants, double the number of infants (n = 660) included in the most recent Cochrane Review. The primary outcome of this trial was a composite of death within seven days or admission to the NICU with moderate-to-severe HIE on days 1 to 5 during hospitalization. LM conferred no benefit over FM for the composite outcome of death or HIE. This trial was conducted in a low-resource setting in Uganda with a high rate (61.2%) of infants born through meconium-stained or foul-smelling amniotic fluid. Approximately 14% of infants in the study needed advanced resuscitation, initiated if a physician was available. Death within 24 h occurred in approximately 30% of infants included in the study. These factors, which inform us about the high-risk nature of the infant population in this trial, have to be considered while interpreting the results.

This accumulating evidence has led to LM use being included in the module on primary PPV in the 8th Edition of the NRP, which is part of NRP Essentials; it now becomes basic airway management training for all providers of neonatal care—not just those expected to be skilled in advanced airway management.

#### 3.8.2. LM as Secondary PPV Interface

The NRP recommends PPV with an alternative airway device if FM ventilation, despite corrective measures, did not result in the desired increasing heart rate response. Four RCTs have been performed so far comparing LM and ETT, of which three made comparisons as a secondary airway device following the failure of resuscitation with FM [58,59,60] (see Table 2). A three-arm study compared LM, ETT, and FM simultaneously as a primary ventilatory device and found that LM is more effective than both FM and ETT during newborn resuscitation [18]. The Cochrane meta-analysis conducted in 2018 included three RCTs with a cumulative sample size of 158. Seven outcomes were systematically compared between the two devices studied. They were (1) failure to correctly insert the device, (2) successful insertion at the first attempt, (3) insertion time, (4) ventilation time, (5) Apgar score ≤ 7 at 5 min, (6) soft tissue trauma after device insertion, (7) death or HIE. Three out of the seven outcomes compared included just one study for analysis. There was no statistical difference in these seven outcomes between LM and ETT. The review found that LM offers comparable efficacy to endotracheal intubation (very low- to low-quality evidence) [13].

Since the completion of the Cochrane review, one RCT has been published, which compared LM and ETT as the secondary airway device [60]. This study included infants with gestational age ≥ 34 weeks and birth weight ≥ 2 kg who required resuscitation with FM PPV for 30 s. This single-center dual-arm randomized trial was conducted in Egypt with a sample size of 80 (40 patients in each arm). The primary outcome studied was the proportion of newborns needing ETT after LM insertion. Secondary outcome measures were Apgar score at 1 and 5 min and O_2_ saturation at 1 and 5 min. There was no need for ETT in all 40 infants randomized to the LM arm. Apgar scores and oxygen saturation at 1 and 5 min were not different. LM insertion was quicker than ETT (9.7 s (3.25) versus 18.08 s (4.8)). Post resuscitation acid-base status showed better mean (SD) pH (7.28 (0.09) versus 7.34 (0.07)) and PaO_2_ (52.74 (13.07) versus 58.39 (10.94)) in the ETT arm compared to LM.

### 3.9. Advantages of LM as a PPV Interface

LM provides relatively stable airway access that is less invasive and can be obtained without laryngoscopy. By avoiding laryngoscopy, we can prevent the potential risks of adverse tracheal intubation-associated events, esophageal intubation, cardiac arrest, endobronchial intubation, airway trauma, laryngospasm, hypotension, and oxygen desaturation [62,63]. LM insertion technique can be easily taught even to novice providers, and the skills can be retained better than both FM and endotracheal intubation [53,64]. LM can be inserted much faster than ETT, with a higher first-attempt success rate. In a comparative randomized trial among experienced providers trained in endotracheal intubation, the time to ventilation was significantly shorter with LM compared to ETT (38.9 ± 1.9 s versus 206.1 ± 31.9 s, *p* < 0.0001) [65].

### 3.10. Disadvantages of LM as a PPV Interface

Aspiration of gastric contents is a known problem with the use of LM. However, the significance of this problem in the context of resuscitation of an asphyxiated newborn in the delivery room has not been studied so far. Compared to ETT, LM does not provide us the ability to suction the trachea if needed. The guidelines regarding managing non-vigorous infants exposed to meconium-stained amniotic fluid with the risk of meconium aspiration have changed in the last decade. The practical relevance of this potential disadvantage of LM has to be studied further. Gastric insufflation is a known problem using first-generation LM, which could not suction the stomach. Second-generation devices such as LM Supreme have an esophageal drain to overcome this problem. I-gel incorporates a gastric channel to vent the stomach; however, size 1 (neonatal size) does not currently have this option. A study conducted in anesthetized adults in laparoscopic surgery compared gastric insufflation with various second-generation LMs and endotracheal tubes and found no apparent difference [66]. A recent report on the use of second-generation LM in a 5-week-old infant supports the claims of the adult studies [67]. LM sizes that are currently available are not generally recommended for preterm infants < 1500 g, but they have been used in VLBW newborns [37] and even in an 800 g neonate [57].

### 3.11. LM during Chest Compressions

AHA/NRP strongly recommends the insertion of an ETT before commencing advanced resuscitation such as chest compression (CC). NRP recommends the use of LM as an alternative if ETT insertion is not feasible. This recommendation is based on the consensus that an invasive PPV interface could provide improved ventilatory efficacy and better coordination between ventilation and chest compressions. However, the feasibility of establishing ETT ventilation in a timely, effective manner before chest compression is a concern shared by experts [68]. With this concern, the European Resuscitation Council—Neonatal Life Support (ERC-NLS) allows the resuscitator to consider ETT or LM before CC without mandating the need for an invasive airway [69]. Nevertheless, ERC-NLS acknowledges that poor delivery of FM ventilation is common and can be suboptimal during CC [70]. LM as an airway device during chest compressions has not been evaluated in neonates. Possible reasons for this lack of evidence are the rarity of the need for CC in neonatal resuscitation at delivery and the infrequent use of LM by the neonatal community for various reasons. In this scenario, NRP recommends that it is reasonable to attempt CC with LM if tracheal intubation is unsuccessful. CC concurrent with SGAD ventilation has been studied in adults in two large RCTs [71,72]. These trials have suggested that SGADs could be the strategy of choice for resuscitating adults with out-of-hospital cardiac arrest by the paramedics, and ETT offers no clinical advantage [73]. This supports NRP’s current recommendation.

There is some emerging evidence regarding using laryngeal masks in delivery room CPR in animal models. Recently reported results of a non-inferiority trial in a lamb model that compared LMA Supreme with ETT in the CPR following cord occlusion induced asphyxial cardiac arrest and found no difference in the primary outcome of time to return of spontaneous circulation (ROSC) [74]. The study also compared the incidence of ROSC, ventilatory pressures utilized, oxygenation, and hemodynamic parameters and found no difference between the two groups.

A large retrospective registry study of neonates receiving CC in the delivery room found wide variability in the bedside application of NRP recommendations. This study found that CCs were initiated before endotracheal intubation in 79% of the study population [75]. The number of attempts at intubation was as high as seven in the group that did not achieve ROSC. The study found that an increased number of intubation attempts was one of the factors independently associated with decreased odds of ROSC. Broader adoption of the current NRP guidelines to utilize LM as an alternative airway may expedite effective ventilation during complicated resuscitations and potentially decrease rescue failures [76].

### 3.12. LM for Medications during Resuscitation

ILCOR and AHA NRP consider epinephrine administration through the ETT as a less effective route. However, considering the delays in establishing venous access during delivery room resuscitation, ETT epinephrine with a relatively higher dose is allowed during the interim [77]. LM has not been evaluated for the administration of epinephrine during neonatal resuscitation. NRP does not recommend the administration of epinephrine through LM. In such a scenario, animal experiments have given some information regarding the feasibility and efficacy of the administration of epinephrine through LM. A study carried out in a porcine model of asphyxial cardiac arrest compared the peak epinephrine concentration administered by three routes: endotracheal tube (ETT), the upper end of the LM, and a catheter inserted through the LM into the trachea (LMC). Peak plasma epinephrine level was lowest with epinephrine administration from the upper end without a catheter. Plasma epinephrine levels were not different between ETT and LMC groups [78]. However, a similar study conducted in an anesthetized porcine model suggested that LM administration of six times the standard tracheal dose of epinephrine may be needed to achieve statistically equivalent hemodynamic changes. However, this experiment was carried out with an endotracheal tube left in situ with the cuff deflated during the administration of epinephrine through the upper end of LM without a catheter [79]. Further studies are needed to understand the efficacy of LM-administered epinephrine during neonatal resuscitation.

LM has been studied more extensively for the administration of surfactant in neonates. Eight RCTs have been conducted so far with an aggregate of 507 newborn infants [37,80,81,82,83,84,85,86]. Surfactant loss into the stomach is usually minimal. However, based on the available clinical evidence with surfactant administration through LM and the above-noted animal research on epinephrine, future studies that intend to evaluate the efficacy of epinephrine administration through LM should use a catheter for drug delivery.

### 3.13. LM Use in Preterm Infants

The manufacturer’s recommendation for the LMA classic and LMA supreme size 1 does not include a lower weight limit with a specified upper limit of <5 kg. I-gel size 1 comes with a manufacturer recommendation of 2–5 kg body weight. The clinical trials that studied LMA classic and LMA Supreme for neonatal resuscitation have used the device in infants with birth weight as low as 1.5 kg, and there is a published report of LM used in an 800 g newborn [57]. The RCTs that have evaluated the LM for surfactant administration have used various types of first- and second-generation supraglottic airway devices in preterm infants with birth weights as low as 1000 g [37,87]. In a small feasibility study to assess LM as a conduit for surfactant administration, preterm infants as low as 28 weeks gestational age and birth weight as low as 880 g have been studied successfully [88]. However, the use of an inappropriate sized LM in preterm infants carries the risk of airway obstruction, gastric insufflation due to inadequate seal, trauma to the oropharynx, upper airway, and esophagus [89].

### 3.14. LM for Difficult Airway

LM is the first-choice ventilation device for infants with difficult airways presenting with an “inability to ventilate and inability to intubate”. Several case reports and case series have described the success of ventilation with LM in infants with syndromes associated with airway anomalies. Pierre Robin sequence, Smith Lemli Opitz syndrome, Treacher Collins syndrome, Cornelia de Lange syndrome, laryngotracheal-esophageal clefts, and congenital centrofacial dysgenesis are some of the reported clinical conditions where LM was successfully used to ventilate or used as an intubating airway [90,91,92,93,94,95,96].

### 3.15. LM Use as an Element of Palliative Care

There are no published studies describing LM use in newborn palliative care. In one of the authors’ practices in a tertiary care NICU in the US, LM has been offered to families and used as an option for a gentler, less invasive airway than an ETT in neonates with poor prognosis (with or without a difficult airway), in whom the family desires only short-term ventilation to allow them some more time with a living newborn. This option has been used both for delivery room resuscitations and for some resuscitations in the NICU setting.

### 3.16. LM Use in Prehospital Settings

Unplanned out-of-hospital deliveries infrequently occur in developed countries, but they carry a high risk of adverse outcomes [97]. Most unplanned out-of-hospital deliveries occur at home, followed by an ambulance or the car en route to the hospital [98]. A study that evaluated the EMS personnel’s comfort level in neonatal resuscitation and NRP compliance in out-of-hospital unplanned deliveries found that 66% of the 230 respondents either never had NRP training or completed NRP training more than two years ago. EMS personnel in this study were neither comfortable with basic skills in the initial stabilization of the infant nor had the newborn size-specific equipment for use [99]. Training EMS personnel for the low-frequency, high-risk scenario should potentially involve skills that have ease of training, high success rate, and low skill decay. LM has a potential role in neonatal resuscitation by EMS both as a primary and secondary PPV device [100].

### 3.17. LM Use in Low Resource Settings

In low-middle income countries (LMIC), the proportion of deliveries attended by skilled and traditional birth attendants which occur outside the health care settings constitute up to 50% of all deliveries occurring in these regions [101]. A systematic review of the acquisition and retention of knowledge and skills by birth attendants in LMICs found that bag-mask ventilation was a difficult skill for birth attendants to learn. Knowledge and skills degrade over time, especially with bag-mask ventilation as a significant barrier to the success of neonatal resuscitation training programs in LMIC [102]. Integrating LM use into the Helping Babies Breathe program could be one of the interventions that possibly improve the neonatal mortality rates further in LMIC [103].

### 3.18. LM Use—NRP 2020

The 8th edition of AAP NRP training is offered as two separate courses (1) NRP Essentials and (2) NRP Advanced. LM has been combined with positive pressure ventilation and included in the NRP Essentials course [38]. This is a shift from the 2015 NRP course framework, which had LM grouped with endotracheal intubation as an alternative airway [20]. This shift underlies the growing evidence that LM could be a safe and effective primary PPV interface, and its use is a desirable skill for all LM providers. This could improve the availability, training, and utilization of LM within the current evidence-based recommendations framework.

### 3.19. Future Directions

Good-quality randomized controlled trials conducted in the last decade have enhanced our understanding of the utility of laryngeal masks. However, many questions need to be answered to bridge our knowledge gaps. First, clinical trials are needed to identify LM models that will be most effective in neonatal resuscitation, easy to insert with a high first-attempt success rate while being cost-effective. Identifying a single device type would standardize the availability and training of the LM in the delivery room resuscitation; however, the range of neonatal airway sizes and anatomical variants will require, at a minimum, an appropriate range of neonatal sizes. Second, future trials comparing LM with FM or ETT should look for outcomes beyond the success of delivery room resuscitation. Outcome variables such as post resuscitative care for perinatal asphyxia, which may be early surrogates for mortality and morbidity, must be measured and compared. Randomized clinical trials are needed to evaluate the feasibility and efficacy of LM ventilation during chest compression. Studies should evaluate the safety of using LM for delivery room resuscitation in the gestational age and birth weight class studied for surfactant administration. Studies should explore whether LM as a conduit for endotracheal intubation has a role in the delivery room resuscitation of newborns. Simulation studies should evaluate better training methods for skill acquisition and retention. Studies aimed at observing the trend in availability and utilization of LM after the 2020 NRP recommendations will inform us better for the future. Preclinical studies are needed to identify protocols for managing infants born through meconium-stained amniotic fluid when LM is used as a primary and secondary airway device.

## 4. Conclusions

This review reiterates the safety and efficacy of LM comparable to FM in the initial stages of neonatal resuscitation for infants ≥34 weeks and ≥1500 g while surveying other potential uses of this type of device. LM may be more effective than FM as a primary ventilation interface in the same population when used by the providers who undertake neonatal resuscitation infrequently. LM is a safe and effective alternative secondary ventilation interface comparable to ETT after the failure of FM ventilation in infants ≥34 weeks. It may be considered the first choice by providers with a perceived lack of confidence in endotracheal intubation.

## Figures and Tables

**Table 1:** Studies comparing LM with FM as the primary PPV interface.

Study	Population	Sample Size (n)	Intervention	Comparator	Primary Outcome (Definition)
Randomized controlled trials					
Singh et al. (2005) [17]	GA > 34 w BW > 1500 g	25 V 25	LMA Classic	FM	Success of ventilation (Chest expansion and bilateral breath sounds) (96% vs. 88%)
Feroze et al. (2008) [18]	BW > 1500 g	25 V 25	LMA Classic	FM	Success of resuscitation (Not clearly defined) (96% vs. 80%)
Zhu et al. (2011) [52]	GA ≥ 34 w BW ≥ 2 kg	205 V 164	LMA Classic	FM	Success of resuscitation (Prevention of need for tracheal intubation) (99% vs. 84.1%)
Mathai et al. (2014) [54]	GA > 36 w BW > 2 kg	32 V 35	LMA Classic	FM	Duration of PPV until spontaneous breathing 95.31 s (23.22 s) vs. 180.86 s (37.83 s) (*p* = 0.024)
Trevisanuto et al. (2015) [19]	GA ≥ 34 w BW ≥ 1500 g	71 V 71	LMA Supreme	FM	Success of resuscitation (Prevention of need for endotracheal intubation) (91.5% vs. 78.9%)
Pejovic et al. (2018) [53]	GA > 34 w BW > 2 kg	25 V 25	i-gel	FM	Time to spontaneous breathing (153 s (59) vs. 216 s (92))
Pejovic et al. (2020) [32]	GA > 34 w BW > 2 kg	563 V 591	i-gel	FM	Death or moderate- severe HIE (27.4% vs. 24.4%)
Observational studies					
Cohort studies					
Trevisanuto et al. (2004) [55]	GA ≥ 34 w BW ≥ 2 kg	74 V 74	LMA Classic	FM	Need for tracheal intubation—no difference
Zanardo et al. (2010) [56]	34w–36 w 7 d	36 V 34	LMA Classic	FM	Admission to NICU [OR—0.30 (0.10–0.89)] and length of hospitalization decreased with LM
Case series					
Paterson et al. (1994) [16]	GA 35–41 w	20	LMA Classic	-	Success of resuscitation—100% (Improvement in APGAR score)
Gandini et al. (1999) [57]	GA 28–42 w BW 1–4.7 Kg	104	LMA Classic	-	Success of resuscitation—99% (Improvement in APGAR score)

**Table 2:** Studies comparing LM and ETT as a secondary PPV interface.

Study	Population	Sample Size (n)	Intervention	Comparator	Primary Outcome (Definition)
Randomized controlled trials					
Esmail et al. (2002) [58]	GA ≥ 35 w BW ≥ 2.5 kg	20 V 20	LMA Classic	ETT	Success of resuscitation (Improvement in APGAR score) (100% in both groups)
Feroze et al. (2008) [18]	BW >1500 g	25 V 25	LMA Classic	ETT	Success of resuscitation (Improvement in APGAR score) (96% vs. 90%)
Yang et al. (2016) [59]	GA ≥ 34 w BW ≥ 2 kg	36 V 32	LMA Classic	ETT	Success of resuscitation (Establishment of spontaneous breathing, HR > 100, good muscle tone) (86% vs. 97%) *p* = 0.20
El-Shimi et al. (2018) [60]	GA ≥ 34 w BW ≥ 2 kg	40 V 40	LMA Classic	ETT	Need for ETT insertion in LMA group (0%) Success of resuscitation (Improvement in APGAR score at 5 min) (100% vs. 100%)
Observational studies					
Cohort study					
Zanardo et al. (2010) [56]	34 w–36 w 7 d	36 V 16	LMA Classic	ETT	Admission to NICU [OR—0.08 (0.02–0.33)] and length of hospitalization decreased with LM
Case-Control study					
Zanardo et al. (2004) [61] (Secondary comparison)	GA > 37 w	43 V 18	LM (Mode of delivery – cesarean section + vaginal)	ETT (Mode of delivery – cesarean section + vaginal)	Success of resuscitation with LM—97.6% Need for NICU admission and mechanical ventilation—decreased in LM group

## References

[1] Wyllie J. (2006). Resuscitation of the depressed newborn. Semin. Fetal Neonatal Med..

[2] Ersdal H.L., Mduma E., Svensen E., Perlman J.M. (2012). Early initiation of basic resuscitation interventions including face mask ventilation may reduce birth asphyxia related mortality in low-income countries: A prospective descriptive observational study. Resuscitation.

[3] Aziz K., Lee H.C., Escobedo M.B., Hoover A.V., Kamath-Rayne B.D., Kapadia V.S., Magid D.J., Niermeyer S., Schmölzer G.M., Szyld E. (2020). Part 5: Neonatal Resuscitation: 2020 American Heart Association Guidelines for Cardiopulmonary Resuscitation and Emergency Cardiovascular Care. Circulation.

[4] Kuypers K.L., Lamberska T., Martherus T., Dekker J., Böhringer S., Hooper S.B., Plavka R., Pas A.B.T. (2019). The effect of a face mask for respiratory support on breathing in preterm infants at birth. Resuscitation.

[5] Martherus T., Oberthuer A., Dekker J., Hooper S.B., McGillick E., Kribs A., Pas A.T. (2019). Supporting breathing of preterm infants at birth: A narrative review. Arch. Dis. Child. Fetal Neonatal Ed..

[6] Singh G.P., Chowdhury T., Bindu B., Schaller B. (2016). Sudden Infant Death Syndrome—Role of Trigeminocardiac Reflex: A Review. Front. Neurol..

[7] Gaertner V.D., Rüegger C.M., O’Currain E., Kamlin C.O.F., Hooper S.B., Davis P.G., Springer L. (2021). Physiological responses to facemask application in newborns immediately after birth. Arch. Dis. Child. Fetal Neonatal Ed..

[8] Schmölzer G.M., Dawson J.A., Kamlin C.O., O’Donnell C.P., Morley C.J., Davis P.G. (2011). Airway obstruction and gas leak during mask ventilation of preterm infants in the delivery room. Arch. Dis. Child. Fetal Neonatal Ed..

[9] Perlman J.M., Risser R. (1995). Cardiopulmonary resuscitation in the delivery room. Associated clinical events. Arch. Pediatr. Adolesc. Med..

[10] Kuypers K.L., Lamberska T., Martherus T., Dekker J., Böhringer S., Hooper S.B., Plavka R., Pas A.B.T. (2020). Comparing the effect of two different interfaces on breathing of preterm infants at birth: A matched-pairs analysis. Resuscitation.

[11] Mangat A.K., Bruckner M., Schmölzer G.M. (2021). Face mask versus nasal prong or nasopharyngeal tube for neonatal resuscitation in the delivery room: A systematic review and meta-analysis. Arch. Dis. Child..

[12] Bansal S.C., Caoci S., Dempsey E., Trevisanuto D., Roehr C.C. (2018). The Laryngeal Mask Airway and Its Use in Neonatal Resuscitation: A Critical Review of Where We Are in 2017/2018. Neonatology.

[13] Qureshi M.J., Kumar M. (2018). Laryngeal mask airway versus bag-mask ventilation or endotracheal intubation for neonatal resuscitation. Cochrane Database Syst. Rev..

[14] Brain A.I. (1983). The laryngeal mask—A new concept in airway management. Br. J. Anaesth..

[15] Mizushima A., Wardall G.J., Simpson D.L. (1992). The laryngeal mask airway in infants. Anaesthesia.

[16] Paterson S.J., Byrne P.J., Molesky M.G., Seal R.F., Finucane B.T. (1994). Neonatal resuscitation using the laryngeal mask airway. Anesthesiology.

[17] Singh R. (2005). Controlled trial to evaluate the use of LMA for neonatal resuscitation. J. Anaesth. Clin. Pharmacol..

[18] Feroze F., Masood N., Khuwaja A., Malik F.I. (2008). Neonatal Resuscitation. Prof. Med. J..

[19] Trevisanuto D., Cavallin F., Nguyen L.N., Nguyen T.V., Tran L.D., Tran C.D., Doglioni N., Micaglio M., Moccia L. (2015). Supreme Laryngeal Mask Airway versus Face Mask during Neonatal Resuscitation: A Randomized Controlled Trial. J. Pediatr..

[20] Weiner G.M., Zaichkin J., Kattwinkel J., American Academy of Pediatrics, American Heart Association (2016). Textbook of Neonatal Resuscitation.

[21] Ramaiah R., Das D., Bhananker S.M., Joffe A.M. (2014). Extraglottic airway devices: A review. Int. J. Crit. Illn. Inj. Sci..

[22] Miller D.M. (2004). A proposed classification and scoring system for supraglottic sealing airways: A brief review. Anesth. Analg..

[23] Wahlen B.M., Heinrichs W., Latorre F. (2004). Gastric insufflation pressure, air leakage and respiratory mechanics in the use of the laryngeal mask airway (LMATM)in children. Pediatr. Anesth..

[24] Ozden E.S., Meco B.C., Alanoglu Z., Alkıs N. (2016). Comparison of ProSeal laryngeal mask airway (PLMA) with cuffed and uncuffed endotracheal tubes in infants. Bosn. J. Basic Med. Sci..

[25] Pejovic N.J., Cavallin F., Mpamize A., Lubulwa C., Höök S.M., Byamugisha J., Nankunda J., Tylleskär T., Trevisanuto D. (2022). Respiratory monitoring during neonatal resuscitation using a supraglottic airway device vs. a face mask. Resuscitation.

[26] Mahdavi A., Razavi S.S., Malekianzadeh B., Sadeghi A. (2017). Comparison of the Peak Inspiratory Pressure and Lung Dynamic Compliance between a Classic Laryngeal Mask Airway and an Endotracheal Tube in Children Under Mechanical Ventilation. Tanaffos.

[27] Reissmann H., Pothmann W., Füllekrug B., Dietz R., Schulte am Esch J. (2000). Resistance of laryngeal mask airway and tracheal tube in mechanically ventilated patients. Br. J. Anaesth..

[28] Natalini G., Rosano A., Lanza G., Martinelli E., Pletti C., Bernardini A. (2003). Resistive load of laryngeal mask airway and ProSeal laryngeal mask airway in mechanically ventilated patients. Acta Anaesthesiol. Scand..

[29] Keller C., Brimacombe J.R., Keller K., Morris R. (1999). Comparison of four methods for assessing airway sealing pressure with the laryngeal mask airway in adult patients. Br. J. Anaesth..

[30] Patel B., Bingham R. (2009). Laryngeal mask airway and other supraglottic airway devices in paediatric practice. Contin. Educ. Anaesth. Crit. Care Pain..

[31] Van Zundert A., Brimacombe J. (2008). The LMA SupremeTM—A pilot study. Anaesthesia.

[32] Pejovic N.J., Höök S.M., Byamugisha J., Alfvén T., Lubulwa C., Cavallin F., Nankunda J., Ersdal H., Blennow M., Trevisanuto D. (2020). A Randomized Trial of Laryngeal Mask Airway in Neonatal Resuscitation. N. Engl. J. Med..

[33] Ankay-Yılbaş A., Başaran B., Üzümcügil F., Akça B., İzgi M., Canbay Ö. (2019). Comparison of i-gel, LMA-supreme, LMA-classic and LMAproseal as conduits of endotracheal intubation in newborns and infants: A manikin study. Turk J. Pediatr..

[34] Goel D., Shah D., Hinder M., Tracy M. (2020). Laryngeal mask airway use during neonatal resuscitation: A survey of practice across newborn intensive care units and neonatal retrieval services in Australian New Zealand Neonatal Network. J. Paediatr. Child. Health.

[35] Mani S., Rawat M. Proficiency of Laryngeal Mask Airway Insertion Skill in NRP Certified Providers. Am. J. Perinatol..

[36] Shah B.A., Foulks A., Lapadula M.C., McCoy M., Hallford G., Bedwell S., DeShea L., Szyld E. Laryngeal Mask Use in the Neonatal Population: A Survey of Practice Providers at a Regional Tertiary Care Center in the United States. Am. J. Perinatol..

[37] Pinheiro J.M., Santana-Rivas Q., Pezzano C. (2016). Randomized trial of laryngeal mask airway versus endotracheal intubation for surfactant delivery. J. Perinatol..

[38] Weiner G.M., Zaichkin J., American Academy of Pediatrics, American Heart Association (2021). Textbook of Neonatal Resuscitation.

[39] Brimacombe J., Berry A. (1993). Insertion of the laryngeal mask airway—A prospective study of four techniques. Anaesth. Intensiv. Care.

[40] Hwang J.-W., Park H.-P., Lim Y.-J., Do S.-H., Lee S.-C., Jeon Y.-T. (2009). Comparison of Two Insertion Techniques of ProSeal™ Laryngeal Mask Airway: Standard versus90-degree Rotation. Anesthesiology.

[41] Koo B.-W., Oh A.-Y., Hwang J.-W., Na H.-S., Min S.-W. (2019). Comparison of standard versus 90° rotation technique for LMA Flexible™ insertion: A randomized controlled trial. BMC Anesthesiol..

[42] Yun M.-J., Hwang J.-W., Park S.-H., Han S.-H., Park H.-P., Kim J.-H., Jeon Y.-T., Lee S.-C. (2011). The 90° rotation technique improves the ease of insertion of the ProSeal™ laryngeal mask airway in children. Can. J. Anesth..

[43] Shyam T., Selvaraj V. (2021). Airway management using LMA-evaluation of three insertional techniques-a prospective randomised study. J. Anaesthesiol. Clin. Pharmacol..

[44] Gandini D., Brimacombe J. (2004). Manikin training for neonatal resuscitation with the laryngeal mask airway. Paediatr. Anaesth..

[45] Belkhatir K., Scrivens A., O’Shea J.E., Roehr C.C. (2021). Experience and training in endotracheal intubation and laryngeal mask airway use in neonates: Results of a national survey. Arch. Dis. Child. Fetal Neonatal Ed..

[46] Konrad C., Schüpfer G., Wietlisbach M., Gerber H. (1998). Learning manual skills in anesthesiology: Is there a recommended number of cases for anesthetic procedures?. Anesth. Analg..

[47] Wood F., Morley C.J., Dawson J.A., Kamlin C.O.F., Owen L.S., Donath S., Davis P.G. (2008). Assessing the effectiveness of two round neonatal resuscitation masks: Study 1. Arch. Dis. Child. Fetal Neonatal Ed..

[48] O’Currain E., Davis P.G., Thio M. (2019). Educational Perspectives: Toward More Effective Neonatal Resuscitation: Assessing and Improving Clinical Skills. Neoreviews.

[49] Wood F.E., Morley C.J., Dawson J.A., Davis P.G. (2008). A respiratory function monitor improves mask ventilation. Arch. Dis. Child. Fetal Neonatal Ed..

[50] Trevisanuto D., Parotto M., Doglioni N., Ori C., Zanardo V., Micaglio M. (2012). The Supreme Laryngeal Mask Airway (LMA): A new neonatal supraglottic device: Comparison with Classic and ProSeal LMA in a manikin. Resuscitation.

[51] Micaglio M., Doglioni N., Parotto M., Zanardo V., Ori C., Trevisanuto D. (2006). Training for neonatal resuscitation with the laryngeal mask airway: A comparison of the LMA-ProSeal and the LMA-Classic in an airway management manikin. Paediatr. Anaesth..

[52] Zhu X.Y., Lin B.C., Zhang Q.S., Ye H.M., Yu R.J. (2011). A prospective evaluation of the efficacy of the laryngeal mask airway during neonatal resuscitation. Resuscitation.

[53] Pejovic N.J., Trevisanuto D., Lubulwa C., Höök S.M., Cavallin F., Byamugisha J., Nankunda J., Tylleskär T. (2018). Neonatal resuscitation using a laryngeal mask airway: A randomised trial in Uganda. Arch. Dis. Child..

[54] Mathai S.S., Adhikari K., Joy A. (2014). Laryngeal Mask Airway as Primary Mode in Neonatal Resuscitation–Does it Reduce Need of Positive Pressure Ventilation?. Pediatr. Res. Int. J..

[55] Trevisanuto D., Micaglio M., Pitton M., Magarotto M., Piva D., Zanardo V. (2004). Laryngeal mask airway: Is the management of neonates requiring positive pressure ventilation at birth changing?. Resuscitation.

[56] Zanardo V., Weiner G., Micaglio M., Doglioni N., Buzzacchero R., Trevisanuto D. (2010). Delivery room resuscitation of near-term infants: Role of the laryngeal mask airway. Resuscitation.

[57] Brimacombe J., Gandini D. (1999). Airway rescue and drug delivery in an 800 g neonate with the laryngeal mask airway. Paediatr. Anaesth..

[58] Esmail N., Saleh M., Ali A. (2002). Laryngeal mask airway versus endotracheal intubation for Apgar score improvement in neonatal resuscitation. Egypt J. Anesthesiol..

[59] Yang C., Zhu X., Lin W., Zhang Q., Su J., Lin B., Ye H., Xiaoyu Z. (2016). Randomized, controlled trial comparing laryngeal mask versus endotracheal intubation during neonatal resuscitation—A secondary publication. BMC Pediatr..

[60] el Shimi M.S., Abusaif I., Khafagy S. (2018). Efficacy of Laryngeal Mask Airway in Neonatal Resuscitation. Egypt J. Hosp. Med..

[61] Zanardo V., Simbi A.K., Savio V., Micaglio M., Trevisanuto D. (2004). Neonatal resuscitation by laryngeal mask airway after elective cesarean section. Fetal Diagn. Ther..

[62] Foglia E.E., Ades A., Napolitano N., Leffelman J., Nadkarni V., Nishisaki A. (2015). Factors Associated with Adverse Events during Tracheal Intubation in the NICU. Neonatology.

[63] Drake-Brockman T.F., Ramgolam A., Zhang G., Hall G.L., von Ungern-Sternberg B.S. (2017). The effect of endotracheal tubes versus laryngeal mask airways on perioperative respiratory adverse events in infants: A randomised controlled trial. Lancet.

[64] Mizumoto H., Motokura K., Kurosaki A., Hata D. (2018). Introduction of laryngeal mask airway in Japan, and its rescue use for newborns. Pediatr. Int..

[65] Reinhart D.J., Simmons G. (1994). Comparison of Placement of the Laryngeal Mask Airway With Endotracheal Tube by Paramedics and Respiratory Therapists. Ann. Emerg. Med..

[66] Ye Q., Wu D., Fang W., Wong G.T.C., Lu Y. (2020). Comparison of gastric insufflation using LMA-supreme and I-gel versus tracheal intubation in laparoscopic gynecological surgery by ultrasound: A randomized observational trial. BMC Anesthesiol..

[67] Dodd K.W., Strobel A.M., Driver B.E., Reardon R.F. (2016). Use of a Supraglottic Airway to Relieve Ventilation-Impeding Gastric Insufflation During Emergency Airway Management in an Infant. Ann. Emerg. Med..

[68] Schwindt J., Schwindt E., Grass B., Schäfer S., Kreth U., Hoffmann F. (2021). Intubation in neonatal resuscitation—Compelling necessity or incalculable risk?. Resuscitation.

[69] Madar J., Roehr C.C., Ainsworth S., Ersdal H., Morley C., Rüdiger M., Skåre C., Szczapa T., Pas A.T., Trevisanuto D. (2021). European Resuscitation Council Guidelines 2021: Newborn resuscitation and support of transition of infants at birth. Resuscitation.

[70] Roehr C.C., Madar J., Morley C.J., Ainsworth S., Ersdal H., Rüdiger M., Skåre C., Szczapa T., Pas A.T., Trevisanuto D. (2021). Reply letter to: Intubation in neonatal resuscitation—Compelling necessity or incalculable risk?. Resuscitation.

[71] Wang H.E., Schmicker R.H., Daya M.R., Stephens S.W., Idris A.H., Carlson J.N., Colella M.R., Herren H., Hansen M., Richmond N.J. (2018). Effect of a Strategy of Initial Laryngeal Tube Insertion vs. Endotracheal Intubation on 72-Hour Survival in Adults with Out-of-Hospital Cardiac Arrest: A Randomized Clinical Trial. JAMA.

[72] Benger J.R., Kirby K., Black S., Brett S.J., Clout M., Lazaroo M.J., Nolan J.P., Reeves B.C., Robinson M., Scott L.J. (2018). Effect of a Strategy of a Supraglottic Airway Device vs. Tracheal Intubation During Out-of-Hospital Cardiac Arrest on Functional Outcome: The AIRWAYS-2 Randomized Clinical Trial. JAMA.

[73] Wang H.E., Benger J.R. (2020). Endotracheal intubation during out-of-hospital cardiac arrest: New insights from recent clinical trials. J. Am. Coll. Emerg. Physicians Open.

[74] Mani S., Gugino S., Helman J., Bawa M., Nair J., Chandrasekharan P., Rawat M. Laryngeal mask ventilation with chest compression during neonatal resuscitation: Randomized, non-inferiority trial in lambs. Pediatr. Res..

[75] Halling C., Raymond T., Brown L.S., Ades A., Foglia E.E., Allen E., Wyckoff M.H., Guerguerian A.-M., Atkins D., Fink E. (2021). Neonatal delivery room CPR: An analysis of the Get with the Guidelines^®^-Resuscitation Registry. Resuscitation.

[76] Vali P., Laskminrusimha S. Laryngeal mask airway: An alternate option for all phases of neonatal resuscitation. Pediatr. Res..

[77] McKinsey S., Perlman J.M. (2016). Resuscitative interventions during simulated asystole deviate from the recommended timeline. Arch. Dis. Child. Fetal Neonatal Ed..

[78] Chen K.T., Lin H.J., Jeng H.W., Lin C.C., Guo H.R. (2008). The pharmacological effect of epinephrine administration via laryngeal mask airway in a porcine model of asphyxial cardiac arrest. Emerg. Med. J..

[79] Liao C.-K., Lin H.-J., Foo N.-P., Lin C.-C., Guo H.-R., Chen K.-T. (2010). Epinephrine administration via a laryngeal mask airway: What is the optimal dose?. Signa Vitae.

[80] Attridge J.T., Stewart C., Stukenborg G.J., Kattwinkel J. (2013). Administration of rescue surfactant by laryngeal mask airway: Lessons from a pilot trial. Am. J. Perinatol..

[81] Sadeghnia A., Tanhaei M., Mohammadizadeh M., Nemati M. (2014). A comparison of surfactant administration through i-gel and ET-tube in the treatment of respiratory distress syndrome in newborns weighing more than 2000 grams. Adv. Biomed Res..

[82] Barbosa R.F., Simões E.S.A.C., Silva Y.P. (2017). A randomized controlled trial of the laryngeal mask airway for surfactant administration in neonates. J. Pediatr..

[83] Roberts K.D., Brown R., Lampland A.L., Leone T.A., Rudser K.D., Finer N.N., Rich W.D., Merritt T.A., Czynski A.J., Kessel J.M. (2018). Laryngeal Mask Airway for Surfactant Administration in Neonates: A Randomized, Controlled Trial. J. Pediatr..

[84] Amini E., Sheikh M., Shariat M., Dalili H., Azadi N., Nourollahi S. (2019). Surfactant Administration in Preterm Neonates Using Laryngeal Mask Airway: A Randomized Clinical Trial. Acta Med. Iran..

[85] Gharehbaghi M., Moghaddam Y.J., Radfar R. (2018). Comparing the Efficacy of Surfactant Administration by Laryngeal Mask Airway and Endotracheal Intubation in Neonatal Respiratory Distress Syndrome. Crescent J. Med. Biol. Sci..

[86] Gallup J.A., MBPinheiro J., Ndakor S.M., Pezzano C. (2021). Randomized Trial of Surfactant Therapy via Laryngeal Mask Airway vs. Brief Tracheal Intubation. Pediatrics.

[87] Roberts C.T., Manley B.J., O’Shea J.E., Stark M., Andersen C., Davis P.G., Buckmaster A. (2021). Supraglottic airway devices for administration of surfactant to newborn infants with respiratory distress syndrome: A narrative review. Arch. Dis. Child.-Fetal Neonatal Ed..

[88] Trevisanuto D., Grazzina N., Ferrarese P., Micaglio M., Verghese C., Zanardo V. (2005). Laryngeal mask airway used as a delivery conduit for the administration of surfactant to preterm infants with respiratory distress syndrome. Biol. Neonate.

[89] Trevisanuto D., Parotto M., Doglioni N., Zanardo V., Micaglio M. (2011). Upper esophageal lesion following laryngeal mask airway resuscitation in a very low birth weight infant. Resuscitation.

[90] Yao C.T., Wang J.N., Tai Y.T., Tsai T.Y., Wu J.M. (2004). Successful management of a neonate with Pierre-Robin syndrome and severe upper airway obstruction by long term placement of a laryngeal mask airway. Resuscitation.

[91] Baraka A. (1995). Laryngeal Mask Airway for Resuscitation of a Newborn with Pierre-Robin Syndrome. Anesthesiology.

[92] Leal-Pavey Y.R. (2004). Use of the LMA classic to secure the airway of a premature neonate with Smith-Lemli-Opitz syndrome: A case report. AANA J..

[93] Bucx M.J., Grolman W., Kruisinga F.H., Lindeboom J.A., Van Kempen A.A. (2003). The prolonged use of the laryngeal mask airway in a neonate with airway obstruction and Treacher Collins syndrome. Paediatr. Anaesth..

[94] Galderisi A., De Bernardo G., Lorenzon E., Trevisanuto D. (2015). i-gel: A new supraglottic device for effective resuscitation of a very low birthweight infant with Cornelia de Lange syndrome. BMJ Case Rep..

[95] Jöhr M., Berger T.M., Ruppen W., Schlegel C. (2003). Congenital laryngotracheo-oesophageal cleft: Successful ventilation with the Laryngeal Mask Airway. Paediatr. Anaesth..

[96] Carenzi B., Corso R., Stellino V., Carlino G., Tonini C., Rossini L., Gentili G. (2002). Airway management in an infant with congenital centrofacial dysgenesia. Br. J. Anaesth..

[97] Gunnarsson B., Smárason A.K., Skogvoll E., Fasting S. (2014). Characteristics and outcome of unplanned out-of-institution births in Norway from 1999 to 2013: A cross-sectional study. Acta Obstet. Gynecol. Scand..

[98] Javaudin F., Hamel V., Legrand A., Goddet S., Templier F., Potiron C., Pes P., Bagou G., Montassier E. (2019). Unplanned out-of-hospital birth and risk factors of adverse perinatal outcome: Findings from a prospective cohort. Scand. J. Trauma Resusc. Emerg. Med..

[99] Huynh T., Bahr N., Harrod T., Guise J.-M. (2020). When Seconds Matter: Neonatal Resuscitation in the Prehospital Setting. Pediatrics.

[100] Berry A.M., Brimacombe J.R., Verghese C. (1998). The laryngeal mask airway in emergency medicine, neonatal resuscitation, and intensive care medicine. Int. Anesthesiol. Clin..

[101] Garces A., McClure E., Espinoza L., Saleem S., Figueroa L., Bucher S., Goldenberg R.L. (2019). Traditional birth attendants and birth outcomes in low-middle income countries: A review. Semin. Perinatol..

[102] Reisman J., Arlington L., Jensen L., Louis H., Suarez-Rebling D., Nelson B.D. (2016). Newborn Resuscitation Training in Resource-Limited Settings: A Systematic Literature Review. Pediatrics.

[103] Niermeyer S., Robertson N.J., Ersdal H.L. (2018). Beyond basic resuscitation: What are the next steps to improve the outcomes of resuscitation at birth when resources are limited?. Semin. Fetal Neonatal Med..

